# On Obstetrical Auscultation

**Published:** 1839-01

**Authors:** 


ART. VIII.—*De Auscultatione Obstetricia. Diss. Inaug.* Auct. A. C. CONRADI.—*Christianiæ*, 1837. 12mo. pp. 66.

*On Obstetrical Auscultation.* By A. C. CONRADI, M.D.—*Christiania*, 1837. 12mo. pp. 66.

THE pages of this dissertation may be conveniently referred to by those in search of an accurate register of the opinions of all writers on obstetric auscultation. Besides this, the details on the phenomena occurring during the act of parturition are sufficiently novel to authorize our devoting to them a share of our space. According to Dr. Conradi, then, immediately before labour commences, the force and sonorousness of the *simple* or *placental* (for he is an advocate, though on no original grounds that we can see, of the theory that fixes their seat in the placenta,) pulsations increase. When the pains commence the beats grow weaker, and appear more distant in proportion to the energy of the former. When the uterine contractions are at their maximum the sounds disappear, returning after their cessation. When the arterial pulse varies during labour, the simple pulsations undergo a corresponding change. In the commencing stage, while the pains are comparatively short and trifling, the frequency of the pulsations is less than at a more advanced period. In the progress of the labour the number of simple beats increases, and towards the close they are more frequent, even in the intervals between the uterine contractions, than previously. The following example will illustrate these points:

In a woman examined before labour the radial pulse was eighty in a minute; at the commencement of the first stage the number of arterial beats and of placental pulsations in each quarter of a minute was as follows: 20, 21, 22, 20; 20, 20, 20, 20; 20, 20, 20, 21; 22, 20, 20, 20; 20, 20, 22, 21; 20, 20, &c. At the end of the first period: 21, 21, 22, 23; 24, 26, 27, 25; 24, 23, 22, 21; 21, 21, 21, 23; 24, 26, 27, 25, &c. At the end of the second and beginning of the third period: 22, 24, 26,

27; 28, 27, 25, 24; 23, 23, 24, 26; 27, 25, 23, 23; 23, 24, 27, 28; 29, &c. In the fourth period, after the rupture of the membranes: 23, 24, 26, 28; 28, 27, 25, 24; 24, 25, 26, 27; 29, 28, 26, 27; 26, 28, 29, 30; 31, 31, &c. The head was now born.

It occasionally happens that the simple pulsations sound weaker rather than stronger as the labour advances. When this occurs, hemorrhage usually appears, and the force of the beats decreases in the ratio of its abundance. After the expulsion of the fœtus the placental pulsation in some instances continues to give out its peculiar blowing and murmuring sound, (especially when the uterus is imperfectly contracted;) and, so long as it retains this character, the secundines are not expelled. In the majority of cases, however, after the birth of the infant, it grows obscure and abrupt, and each beat is separated by a distinct interval from those following and preceding it. With these characters, the simple pulsation often continues to exist after the expulsion of the placenta, and until the uterus recovers its ante-gravid condition. The *double* or *fœtal* pulsations also suffer changes during parturition. Sometimes their situation alters, and they appear in a different place from that they had occupied before the pains. When the pain ceases they either retain their new or return to their former position. This peculiar change of place is not observed after the descent of the head into the cavity of the pelvis; the spot from which the sounds issue thenceforth manifestly descends along with the fœtus itself. Their force and frequency are greatly increased at the commencement of labour, to such an extent that from 160 to 170 double beats are not uncommonly noted in a minute. When the head enters the cavity of the pelvis their number is little affected. Dr. Conradi has never observed the sudden decrease from 150 to 90 beats, stated by Thomas to take place at that period of the head's progress, nor found it mentioned by any author. The statement of Thomas, however, seems to receive some support from the fact ascertained by M. Lediberder, and which is noticed in another place, that the mean number of double pulsations is so few as 83·3 during the first minute after birth. After the rupture of the membranes and escape of the liquor amnii, the fœtal pulsations always struck the ear with augmented force. If they grow weak and intermittent, the fœtus is in a state of threatened asphyxia.

